# Left Distal Radial Artery Access Site in Primary Percutaneous Coronary Intervention: Is It Safe?

**DOI:** 10.4274/balkanmedj.galenos.2020.2020.4.49

**Published:** 2020-08-11

**Authors:** Elton Soydan, Mustafa Akın

**Affiliations:** 1 Department of Cardiology, Ege University School of Medicine, İzmir, Turkey

**Keywords:** Acute myocardial infarction, left distal artery, primary percutaneous intervention

## Abstract

**Background:**

Left distal radial artery access site has emerged as a new technique for coronary angiography procedures.

**Aims:**

We aimed at assessing its applicability as an alternative way for primary percutaneous coronary interventions in ST-elevation myocardial infarction.

**Study Design:**

Retrospective observational cohort study.

**Methods:**

Left distal radial artery was used as an access site in 30 consecutive ST-elevation myocardial infarction patients for primary coronary intervention. It was used by experienced operators who were unaware of the study. All patients had a prominent pulse in their left forearm and distal radial artery. Each patient’s left arm was gently bent into his/her right groin with comfortable position of the hand. The operator/s stood at the right side of the patient where both could make the arterial puncture. Demographic features and complications were recorded during the hospital stay.

**Results:**

Mean age of patients was 58 years with a male gender predominance of 87%. Fifteen patients were diagnosed of Inferior elevation myocardial infarction, 14 patients of Anterior, and one of Lateral elevation myocardial infarction. The most common culprit artery was the left anterior descending coronary artery (14 patients). Six patients were in KILLIP class II on admission and only one with Anterior elevation myocardial infarction was in severe pulmonary edema (KILLIP III) during intervention. All the procedures were successfully contemplated with 6 French Judkins catheters. Brachial spasm occurred in one patient which was resolved with intra-arterial nitrate. Transfemoral approach was changed to left distal radial access in 4 patients due to severe bilateral iliac artery disease. Mean puncture time was 37.36 seconds. There was no radial occlusion, hematoma, hand neurologic deficit or bleeding. Patients were discharged on an average duration of 4.2 days.

**Conclusion:**

Left distal radial artery can be used as an alternative safe and feasible access site for successful primary coronary interventions provided that it is performed by experienced operators.

The major advantages of the transradial approach are the reductions of vascular complications and immediate post procedural mobilization. These advantages provided robust evidence favoring the radial route as the default access site in ST segment elevation myocardial infarction (STEMI) patients undergoing primary percutaneous coronary intervention (PCI) (1,2,3,4). Many studies have used the right radial artery as the radial access site. Left radial artery as well is an appropriate alternative access for coronary catheterization and poses less anatomical variations like tortuosity than the right radial artery (5). However, bending of the operator over the left part of the patient to make the puncture into the left radial artery could be a disadvantage and really unpleasant especially in obese patients perhaps making catheterization crossover to another access site. An alternative way to have a more comfortable position for both the patient and the operator is the left distal radial (LDR) artery located in the fossa radialis or the so called “anatomical snuffbox” on the dorsal side of the hand. It can be palpated as the thumb is extended outwards the other digits where the tendons of abductor pollicis longus and extensor pollicis brevis muscles on the palmar side, and the tendon of extensor pollicis longus muscle on the dorsal side become prominent and form the depression triangular area “the fossa radialis” (Figure 1) (6,7). There are few studies showing the convenience of the LDR artery access site for coronary angiography but evaluation of its use in primary PCI is low. As experienced radial operators, our aim is to show the feasibility of this new access site in primary PCI in STEMI patients.

## MATERIALS AND METHODS

**Figure 2:**
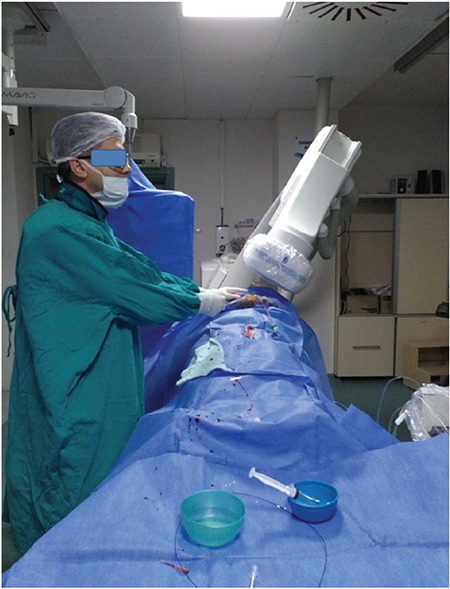
Primary coronary intervention in a patient with Inferior ST segment elevation myocardial infarction. Operator standing at the right of the patient far from the radiation beam.

Primary PCI through the LDR artery was performed in 30 patients consecutively admitted in the radial operator’s duty between January and June 2019 at our hospital. All cases were performed by two experienced LDR operators having performed 500 diagnostic and interventional coronary procedures in the last two years. Other STEMI patients intervened through the femoral approach were not included in the study. Non-STEMI and or unstable angina pectoris patients admitted during this period were excluded. Our study was retrospectively designed in order to get a transparent evaluation in this access site for this special group of patients. All the past relevant data were thoroughly collected from each patient’s electronic recordings. The study got approval by the Local Ethics Committee (protocol no: 19-11.1T/51). All patients had a palpable pulse over their left anatomic snuffbox ‘fossa radialis’. After informed consent, patients were immediately transferred to angiography laboratory for primary PCI. Their left hand was bent over the abdomen near to their right groin and the thumb taken underneath the other four fingers to make the fossa radialis more prominent. The operator stood at the right side of the patient and after disinfection with povidine iodine the patient’s body was covered with sterile clothes with an opening area over the LDR access site (Figure 2). In order to better feel the pulse, local anesthetic with 2 mL of 2% Prilocaine was administered over the AS area after successful puncture with 21 Gauge open needle. Successful puncture was followed by an insertion of 0.018-inch straight guidewire. Then a 6 French radial hydrophilic sheath (Prelude Ease, Merit Medical) was introduced into the LDR artery and a cocktail of 2500 units of unfractionated heparin, 100 mcg of nitrate and 10 mL of saline was administered to all patients to prevent radial spasm. The total required dose of heparin was adjusted intravenously according to patient’s body weight. Six French Judkins catheters were used for all patients with the left guiding catheters having a 3.5 cm of curvature to easily engage into the left main coronary ostium. All catheters were firstly flushed and wiped with wet gauze to facilitate advancement through the upper stream arteries. They were then advanced by a 0.35 inch J tipped wire assistance. Right or left Judkins catheter exchange was achieved by the 0.35 Inch J tipped wire in order to prevent any vessel harm. At procedure termination, the radial sheath was pulled out and early hemostasis was obtained by compression with the thumb of the assistant for approximately 15 minutes then a slightly compressive bandage with gauze was applied over the access site for one day. The left radial artery pulse at the forearm and at AS were checked by palpation after the procedure and during hospitalization.

### Statistical analysis

All the data were analyzed in the SPSS 25 program where continuous and categorical variables were shown as mean ± standard deviation and frequency with proportion respectively.

## RESULTS

Demographic and procedural features are depicted in Table 1 and Table 2 respectively. There was a young population with a mean age of 58 years and male gender predominance of 87%. All patients had a bounded pulse in their AS area palpated by experienced operators. The most common risk factors were hypertension and smoking. Twenty-two patients were diagnosed with newly onset coronary artery disease despite the other 8 having a chronic history of coronary events. Fifteen patients were diagnosed with inferior STEMI with the right coronary artery being the most intervened (11 patients) followed by the left circumflex artery (2 patients). After a successful stent implantation, one of the Inferior STEMI patients experienced spasm in the brachial artery region during pulling out of the catheter. This was resolved by intra-arterial 100 mcg of nitrate. The last 2 patients with Inferior STEMI diagnosis had multivessel disease with extensive thrombotic lesions in their partially opened right coronary artery. Abciximab infusion provided a satisfactory hemodynamic status with resolution of chest pain and ST segment elevation that no further control angiography was required and thus referring them for elective coronary bypass surgery. Fourteen patients had anterior STEMI and the last one had an ST segment elevation in the lateral derivations where a dominant occluded first diagonal artery was successfully intervened. Six patients (5 - anterior; 1 - inferior) had acute heart failure on presentation and were classified as KILLIP class II with rales <50% of lungs. One patient with Anterior STEMI had frank pulmonary edema (KILLIP III: broad fine rales in >50% of lungs area) on admission and was successfully intervened with direct stent procedure on the left anterior descending coronary artery. Interestingly, 7 patients had peripheral artery disease, 4 of whom diagnosed with inferior STEMI had an immediate crossover access site from the right femoral artery to the LDR access because of bilateral severe iliac artery disease with right side total occlusion. Twenty-eight patients had successful primary coronary intervention (93.3%), 6 of whom had a direct stenting procedure. All implanted stents were Zotarolimus drug eluting stents. There was no stent thrombosis and the thrombolysis in myocardial infarction flow was totally restored in the culprit coronary artery. One patient with anterior STEMI had a rescue PCI after intravenous thrombolysis. Mean puncture time defined as the time interval from needle puncture into the left Snuffbox area to successful sheath introduction was 37.36±17.6 seconds and door to balloon time was 27.77±6.23 minutes. Fluoroscopy time was 10.12±5.14 minutes. Radiation exposure expressed by total air kerma and total dose area product was recorded as 917.9±677 mGy and 7723±4037 μGy∙m2 respectively. Study population had a mildly reduced left ventricle ejection fraction (42.1%±7.1%) and no mechanical complication or severe valve dysfunction occurred. All patients took loading doses of 600 mg of Clopidogrel and 300 mg of Aspirin with concomitant weight adjusted heparin. Hospitalization went uneventfully without any vessel related complications such as forearm radial artery occlusion hematoma or access site bleeding. Left radial artery pulse was found in a bounding strength (grade 4) at the forearm and at the fossa radialis both after complete hemostasis and on discharge day. No neurologic deficit of the hand was noticed. Early mobilization of patients was a great valuable sign for their comfort and treatment compliance with a mean hospital stay of 4.2 days.

## DISCUSSION

The LDR artery recently has become famous by showing its feasibility as an alternative access site in coronary angiography. Because of its radial-aortic course resembling that of the femoral-aortic one, the LDR artery has been shown compatible for coronary catheterization. Moreover, the LDR artery lies in a compact triangular area that its superficial course makes the puncture easier than a forearm main radial artery. This hypothesis stimulated us to intervene with this access site and a short puncture time (mean 37.36±17.6 seconds) encouraged us to continue with primary coronary intervention. Importantly, the valuable publication of Babunashvili and Dundua has to be underscored. They were the first to use this technique in the early 90’s with a first publication in 2011 (8). They accurately defined two parts of the LDR artery that was divided by the tendon of the extensor pollicis longus muscle: the base (formed by distal radius, scaphoid, trapezium, and the base of the first metacarpal bone) and the dorsum of the hand respectively (9). Although stated that puncture into the dorsum of the hand is easier, we were more familiar with the base AS area after having a two-year experience with totally 500 elective coronary angiography and intervention cases. Getting practice and experience in elective patients encouraged us to use it in primary PCI. It has to be highlighted the fact that unstable patients with acute heart failure presentation (KILLIP II and III) had a good outcome because of fast puncture access site and no challenge in catheter engagement into the coronary ostia with proper balloon and or stent procedure. Although not objective, visual analog scale was low and the only disturbing point was the insertion of the radial sheath pressing the surrounding vascular and neural tissue. No discomfort was felt by the patients after that. We did not encounter any friction or spasm sign during advancement of catheters because the lumen of the catheters was continuously flushed and surfaces wiped with wet gauze before insertion into the sheath. This was done in order to reduce the friction with upstream artery endothelium and so preventing spasm. All these maneuvers for preventing spasm have accounted for shorter door to balloon times (27.77±6.23 minutes) and implying the applicability of this new technique for successful primary PCI. Although the study population was small, there was no marked vessel tortuosity leading to crossover to another access site but instead, we had 4 patients with non-accessible femoral arteries due to iliac artery occlusions in whom primary PCI was performed by the LDR route. The easy puncture into the AS area and successful contemplation of intervention during this stressful period should emphasize the ready use of this site in terms of severe peripheral artery disease. Our 4 patients’ iliac artery occlusions were successfully treated percutaneously with stent implantation during the hospital stay. Mean fluoroscopy time was little higher in contrast with the RIVAL sub study analysis (10.12 vs 9.3 minutes). However, air kerma radiation exposure was found lower (917.9 versus 1272 mGy) than that of the RIVAL radiation sub study analysis (10). It should be specified that 4 patients with peripheral artery disease crossover to the LDR artery access site and 4 others with prior coronary bypass surgery could have accounted for increased fluoroscopy time. Easy hemostasis without any access site bleeding and or occlusion poses this access site a good alternative for safe management of STEMI patients. Being a distal branch of the main radial artery and multiple collaterals between the superficial and the deep palmar arch makes this site safe against digital ischemia. No radial occlusion supported these protective anatomic features implying that the main radial artery could still be a good alternative for coronary bypass graft and an appropriate access site for opening of arterio-venous fistula occlusion.

The most important limitation of this study is its small population size and its retrospective feature. Instead, acute management of STEMI patients by this new technique is not thoroughly evaluated elsewhere except for one retrospective study newly published supporting our findings and the number of patients studied is thought to be sufficient to make a demonstration of the feasibility and applicability of this approach in consecutive patients in acute and stressful primary coronary intervention procedure (11). Although LDR artery has a small diameter, puncture can be challenging. We noticed that being superficial and located in a compact triangular area puncture can be easier than the forearm main radial artery; the relevant feature that encouraged us to continue with the intervention. Fast hemostasis provided by manual compression was another protective feature in terms of access related vascular complications. Interestingly no patient complained of any ischemic complications. Although not confirmed by Doppler ultrasonography, patients pulse check were normal after the intervention. Comfortability of both patient and operator during the procedure made this technique confident in succeeding primary coronary intervention.

In conclusion, this representative study showed that the LDR artery is a safe alternative site in primary PCI. However, this new access site merits more evaluation in the future and randomized controlled trials in comparison with other (conventional radial and or femoral) access sites will accurately show its deserved place in the acute management of STEMI patients.

## Figures and Tables

**Table 1:**
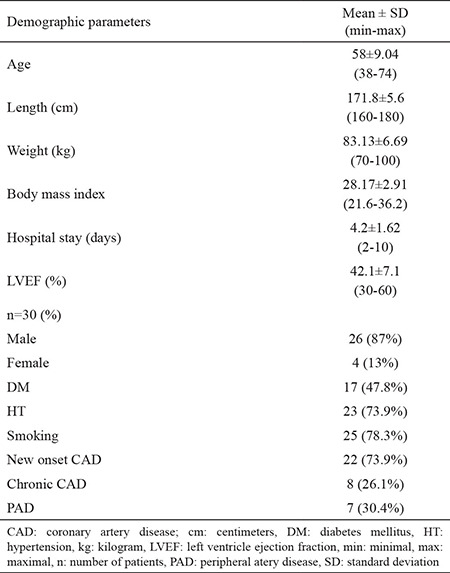
Demographic features of study population (n=30)

**Table 2:**
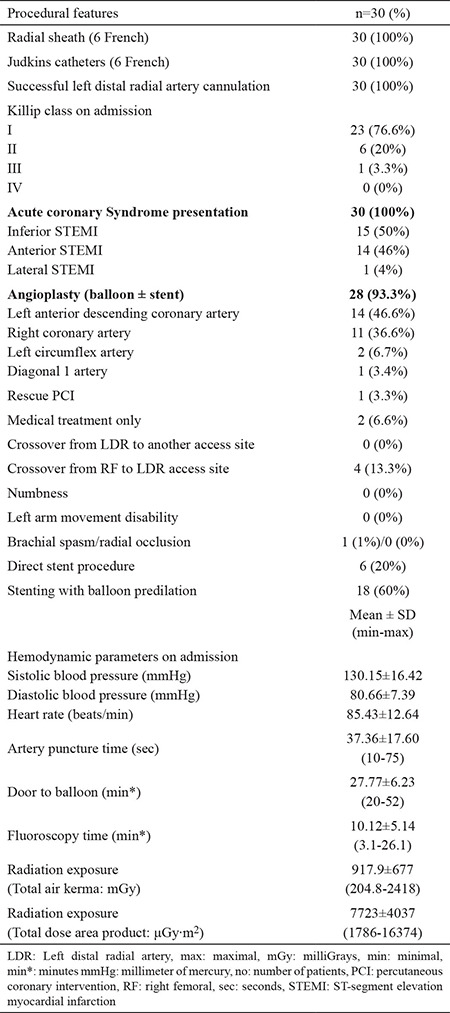
Procedural features of study population

**Figure 1:**
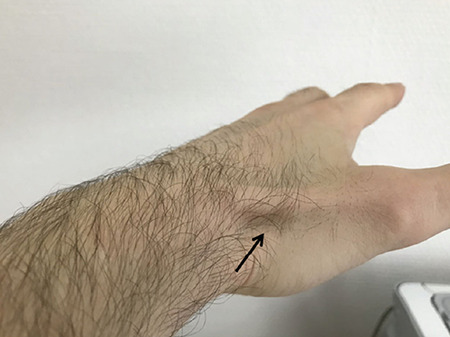
The left anatomical snuffbox (triangular area depicted by black arrow) surrounded on the palmar side by the tendons of abductor pollicis longus and extensor pollicis brevis muscles, and on the dorsal side by the tendon of extensor pollicis longus muscle.
